# Evaluation of the user experience for a point of care molecular test for causes of vaginitis

**DOI:** 10.1186/s12879-025-11304-8

**Published:** 2025-08-02

**Authors:** Barbara Van Der Pol, Rebecca Lillis, Irving Nachamkin, Stephen Young, LaShonda Crane, Jacqueline Brown, R. Lamar Parker, Alice Weissfeld, Xiaohong Liu, Christina Huang, Aarthi Raman

**Affiliations:** 1 University of Alabama at Birmingham, Birmingham, AL US; 2 Louisiana State University Health Science Center, New Orleans, LA US; 3 Department of Pathology and Laboratory Medicine, Perelman School of Medicine, University of Pennsylvania, Philadelphia, PA US; 4 TriCore Reference Laboratories, Albuquerque, NM US; 5 Planned Parenthood Gulf Coast, Houston, TX US; 6 Clinical Trial Network, Houston, TX US; 7 Unified Women’s Clinical Research, Winston Salem, NC US; 8 Microbiology Specialists, Inc, Houston, TX US; 9 Cepheid Inc, Sunnyvale, CA US

**Keywords:** Bacterial vaginosis, Candidiasis, Molecular diagnostics, Point of care diagnostics, Trichomoniasis, Vaginitis

## Abstract

**Background:**

Vaginitis is a major cause of healthcare visits in the US, costing over $1.2 billion annually. Point-of-care (POC) nucleic acid amplification tests (NAATs) could improve accuracy of diagnosis and treatment during initial visit for vaginitis compared to send-out NAATs.

**Methods:**

A secondary analysis of data was collected from test operators at POC testing sites where the Xpert Xpress Multiplex Vaginal Panel test (“MVP test”) was performed. Users completed a survey assessing their experience with the instrument system and the PCR-based MVP test.

**Results:**

The MVP test demonstrated consistent performance, measured by positive and negative percent agreement, across all user categories, including both trained and untrained staff. Performance was evaluated based on job function (clinicians, non-clinicians, support staff) and educational level (bachelor’s degree or higher, associate’s degree or some college, high school diploma/general educational development (GED) with technical certification), with no significant differences in performance (P >0.082 and P >0.050, respectively).

User feedback from 19 operators showed that 15/19 (79%) of users found the GeneXpert Xpress Instrument System easy to set up, and 18/19 (96%) found the test instructions easy to follow. Users described the system as user-friendly with clear instructions and helpful videos. Overall, 19/19 (100%) agreed that the MVP test was easy to perform.

**Conclusions:**

Availability of accurate POC diagnostics will only be useful if the test can be performed by any potential user. Our study results suggest that the MVP test was acceptable to users and that results were accurate regardless of user qualifications. Thus, this test has the potential to improve the immediate clinical outcome by supporting accurate, same day treatment for causes of vaginitis.

**Supplementary Information:**

The online version contains supplementary material available at 10.1186/s12879-025-11304-8.

## Contributions to the literature

- POC NAAT-based diagnostic tests, especially those with Clinical Laboratory Improvements Amendment (CLIA)-waived designation, can reduce the time from sample collection to patient management, improve same-day treatment accuracy, and lower long-term healthcare costs.
- The Xpert Xpress MVP test is the first CLIA-waived, FDA-cleared POC NAAT for diagnosing vaginal infections within an hour with minimal hands-on time.
- The article demonstrated the ease-of-use for operators with varying skill levels for effective implementation of the POC NAAT.

## Introduction

Vaginitis is one of the leading causes of clinical healthcare visits in the United States (US) with estimates of the healthcare burden exceeding 10 million visits at a cost of >$1.2 billion per year [1]. Current testing options include clinical evaluation, microscopy, a DNA probe assay and nucleic acid amplification testing (NAAT), performed as a send-out test. Use of NAATs has been shown to reduce long-term follow up healthcare costs for women initially presenting with vaginitis complaints [2]. However, women with vaginitis are seeking treatment at the time of the visit and thus reliance on laboratory-based send-out NAATs is sub-optimal for immediate care and patient satisfaction. A solution to this problem is to utilize a NAAT test at the point-of-care (POC) where care is delivered directly to the patient, in contrast to testing at centralized laboratories. In this way, clinical evaluation and appropriate and accurate treatment can be provided during the initial visit. Implementation studies of POC tests for detection of chlamydia and gonorrhea have been conducted in settings outside the US and have found that the user experience is a key factor in the successful adoption of this testing modality [3]. 

A multi-site, prospective, method comparison clinical study was conducted to assess the clinical performance of the Xpert Xpress Multiplex Vaginal Panel (Cepheid, Sunnyvale, CA) test (“MVP test”), a new POC NAAT that reports results for Bacterial Vaginosis (BV), Candida group (including C. *albicans*, C. *tropicalis*, C. *parapsilosis*, and C. *dubliniensis*), *Candida glabrata and Candida krusei* (Candida glab-krus), and *Trichomonas vaginalis* (TV). The performance evaluation was the first to extend beyond POC evaluations of chlamydia and gonorrhea, and the results are presented elsewhere [4]. 

Here we describe a secondary data analysis focused on user characteristics and feedback collected at POC sites as part of this study to help understand the user experience of this new POC NAAT test that determines the common causes of vaginitis.

## Materials and methods

### Study design and setting

Collection sites included in this secondary analysis were POC testing environments where testing was performed at the clinical site. These sites included family planning, sexual health, OB/GYN, and women’s health clinics. The study protocol was reviewed and approved by each participating site’s institutional review board (IRB) prior to any study activities. Of the 9 sites, Advarra served as the central IRB at 7 and the other 2 utilized institutional IRBs.

The MVP test is an automated in vitro diagnostic test for qualitative detection of DNA targets from anaerobic bacteria associated with bacterial vaginosis, Candida species associated with vulvovaginal candidiasis, and *Trichomonas vaginalis*, the causative agent of trichomoniasis. The MVP testing was performed within 24 h following collection at POC sites. As part of the evaluation of a Clinical Laboratory Improvement Amendments (CLIA)-waived test, users testing on the GeneXpert Xpress Instrument System (“Xpress System”) were provided with the investigational Instructions For Use (IFU) and Quick Reference Instructions (QRI, see Supplemental Material 1) but were not provided with any additional training (e.g., written or verbal training, coaching, or prompting) on the Xpress System or the MVP test. Additionally, users were instructed not to discuss the test with other users or otherwise coach or observe each other, and they were not allowed to communicate with the manufacturer. Results were for study purposes only and not used for patient management.

### Participants and specimens

A total of nine POC sites prospectively obtained one self-collected (in a clinical setting) vaginal swab (SVS) and five clinician-collected vaginal swab (CVS) specimens from female patients ≥ 14 years of age who presented with signs and/or symptoms of vaginitis/vaginosis (including abnormal vaginal discharge; dysuria; vulvar/vaginal itching, burning, irritation, pain or vulvar edema; coital pain; or vaginal odor). A total of 22 users (5 clinicians, 13 non-clinicians and 4 support staff) across the nine POC sites tested the specimens with the Xpress System.

All users were asked to complete a survey-based assessment of their experiences with performing the assay. A 19-item questionnaire (Supplemental Table 2) was administered to assess the user’s experience related to ease of handling of the Xpress System (unpacking, setup, and assembly), as well ease of performing MVP testing and interpreting test results (see *Supplementary Attachment*). Users were asked to rate their experience based on a Likert scale of 1–5 anchored with 1 being ‘strongly disagree’ is easy and 5 being ‘strongly agree’. Of the 22 POC users, 19 completed the survey and their results are included in the analyses.

### Statistical methods

Questionnaire responses were summarized using descriptive statistics. Performance by user type was assessed by positive percent agreement (PPA) and negative percent agreement (NPA) along with 95% confidence intervals for each subgroup. Differences in overall agreement between subgroups was evaluated using the Fisher’s Exact test to assess differences between user types based on their roles at the testing sites and their educational status. All statistical analyses were performed using R studio.

## Results

### MVP performance by user type

**Fig. 1:**
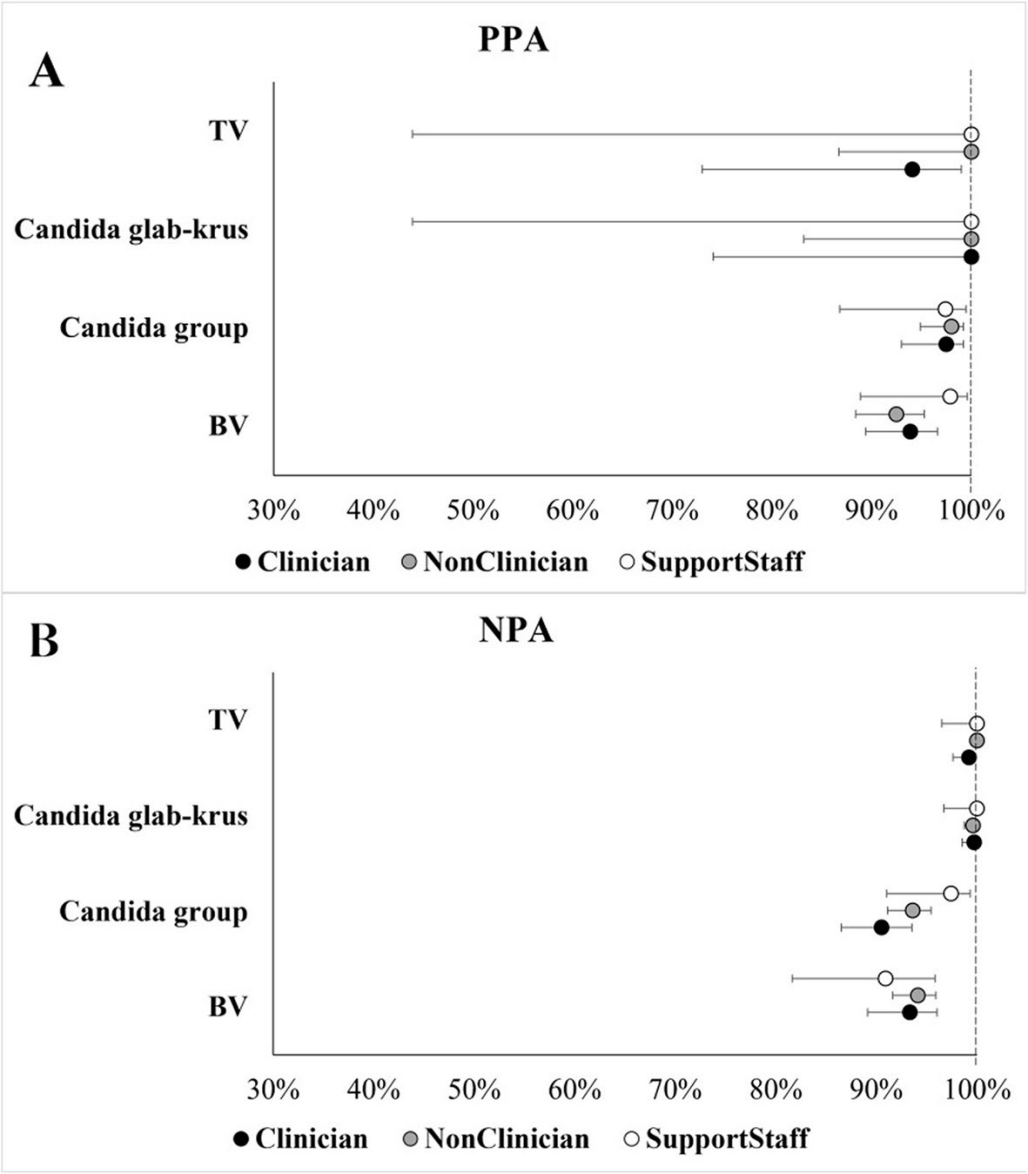
Forest Plot with Point Estimates and 95% Confidence Intervals for PPA (Panel A) and NPA (Panel B) in Self-collected Vaginal Swabs based on the Education Level of the Operator Performing the Test

As previously described, the MVP test demonstrated a high PPA and NPA with the comparator methods for the three causes of vaginitis/vaginosis [4]. Performance was consistent across all categories of users in the parent study where some testing was performed on-site by untrained users and other testing was performed by trained staff in laboratory settings. Clinical performance was evaluated across user types categorized based on their job functions within the testing site: clinicians (e.g. physicians, other advanced practice providers and nurses), non-clinicians (e.g. medical assistants and clinical specialists) and support staff (e.g. clinic assistants and clinical managers). Clinical performance was also evaluated by categorizing users based on their educational levels (Level 1 = bachelor’s degree or higher; Level 2 = associate’s degree or some college level courses; Level 3 = high school diploma/General Educational Development (GED) with some technical certification) (Fig. 1).

**Fig. 2:**
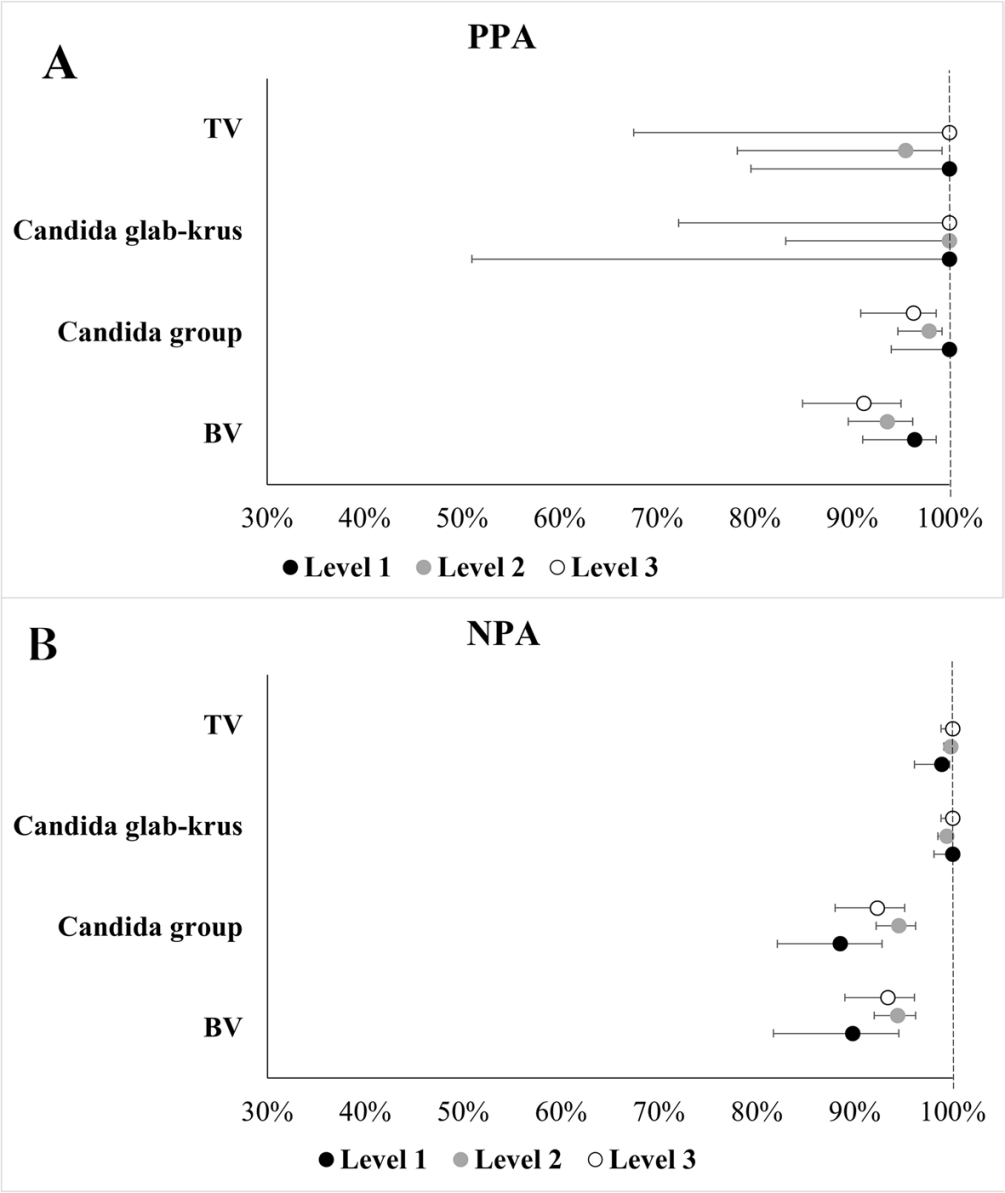
Plot with Point Estimates and 95% Confidence Intervals for PPA (Panel A) and NPA (Panel B) in Self-collected Vaginal Swabs based on the Type of Operator Performing the Test

Based on PPA and NPA estimates for BV, TV, Candida spp. and Candida glab-krus results of the MVP test, there were no statistically significant differences in performance between user types (*P* > 0.082 across these subgroups) (Table 1; Fig. 2) nor in performance based on users’ educational status (*P* > 0.050 across various subgroups) (Table 2; Fig. 2) despite wide confidence intervals for both TV and Candida glab-krus results. Also, the test showed high performance and was not affected by vaginal sample self-collection by patients (Supplemental Table 1). Given the small sample sizes of support-staff and Level 3 cohorts, further studies with larger sample sizes would be needed to draw more reliable conclusions.

**Table 1:** Clinical performance in Self-Collected vaginal swab specimens across user types

Target	User Type	Positive	Positive Agreement (*N*)	Positive Agreement (%)	95% CI	Fisher’s Exact *p*-value for PPAs	Negative	Negative Agreement (*N*)	Negative Agreement (%)	95% CI	Fisher’s Exact *p*-value for NPAs
BV	Clinician	180	169/180	93.9	89.4–96.6	0.454	210	196/210	93.3	89.1–96	0.576
	Non-clinician	237	219/237	92.4	88.3–95.1		484	455/484	94	91.5–95.8	
	Support Staff	47	46/47	97.9	88.9–99.6		66	60/66	90.9	81.6–95.8	
TV	Clinician	17	16/17	94.1	73–99	0.413	368	365/368	99.2	97.6–99.7	0.082
	Non-clinician	28	28/28	100	87.9–100		689	689/689	100	99.4–100	
	Support Staff	3	3/3	100	43.9–100		105	105/105	100	96.5–100	
CS	Clinician	121	118/121	97.5	93–99.2	0.785	275	249/275	90.5	86.5–93.5	0.090
	Non-clinician	207	203/207	98.1	95.1–99.2		513	480/513	93.6	91.1–95.4	
	Support Staff	39	38/39	97.4	86.8–99.5		77	75/77	97.4	91–99.3	
CgCk	Clinician	11	11/11	100	74.1–100	N/A	385	384/385	99.7	98.5–100	1.000
	Non-clinician	19	19/19	100	83.2–100		701	698/701	99.6	98.7–99.9	
	Support Staff	3	3/3	100	43.9–100		113	113/113	100	96.7–100	

*BV *Bacterial vaginosis, *CI *Confidence interval, *CS *Candida species, *CgCk *Candida glabrata/Candida krusei, *TV *Trichomonas

**Table 2:** Clinical performance in Self-Collected vaginal swab specimens categorized based on user’s educational status

Target	Levels	Positive	Positive Agreement (*N*)	Positive Agreement (%)	95% CI	Fisher’s Exact *p*-value for PPAs	Negative	Negative Agreement (*N*)	Negative Agreement (%)	95% CI	Fisher’s Exact *p*-value for NPAs
BV	1	111	107/111	96.4	91.1–98.6	0.251	88	79/88	89.8	81.7–94.5	0.255
	2	219	205/219	93.6	89.6–96.2		466	440/466	94.4	92–96.2	
	3	134	122/134	91	85–94.8		206	192/206	93.2	88.9–95.9	
TV	1	15	15/15	100	79.6–100	1.000	183	181/183	98.9	96.1–99.7	0.121
	2	22	21/22	95.5	78.2–99.2		655	654/655	99.8	99.1–100	
	3	11	11/11	100	74.1–100		324	324/324	100	98.8–100	
CS	1	60	60/60	100	94–100	0.354	139	123/139	88.5	82.1–92.8	0.050
	2	189	185/189	97.9	94.7–99.2		495	468/495	94.5	92.2–96.2	
	3	118	114/118	96.6	91.6–98.7		231	213/231	92.2	88–95	
CgCk	1	4	4/4	100	51–100	N/A	195	195/195	100	98.1–100	0.382
	2	19	19/19	100	83.2–100		665	661/665	99.4	98.5–99.8	
	3	10	10/10	100	72.2–100		339	339/339	100	98.9–100	

*BV *Bacterial vaginosis, *CI *Confidence interval, *CS *Candida species, *CgCk *Candida glabrata/Candida krusei, *TV *Trichomonas

Level 1 = bachelor’s degree or higher; Level 2 = associate’s degree or some college level courses; Level 3 = high school diploma/GED with some technical certification

### User experience

Based on input from 19 operators who participated in the clinical study, majority (15/19, 79%) of users rated that they ‘agree/strongly agree’ to one of three questions on the ease of Xpress System setup, while 4/19 (21%) rated a ‘neutral’ score. Of the questions that evaluated the ease of running a test, 18/19 (96%) of the responses indicated that users ‘agree/strongly agree’ that the instructions to load the sample into the cartridge and place it into the instrument for the MVP test in the QRI were easy to follow. More than 90% of the responses from users indicated that they “agree/strongly agree” that the repeat test instructions in the QRI were easy to follow.

**Fig. 3:**
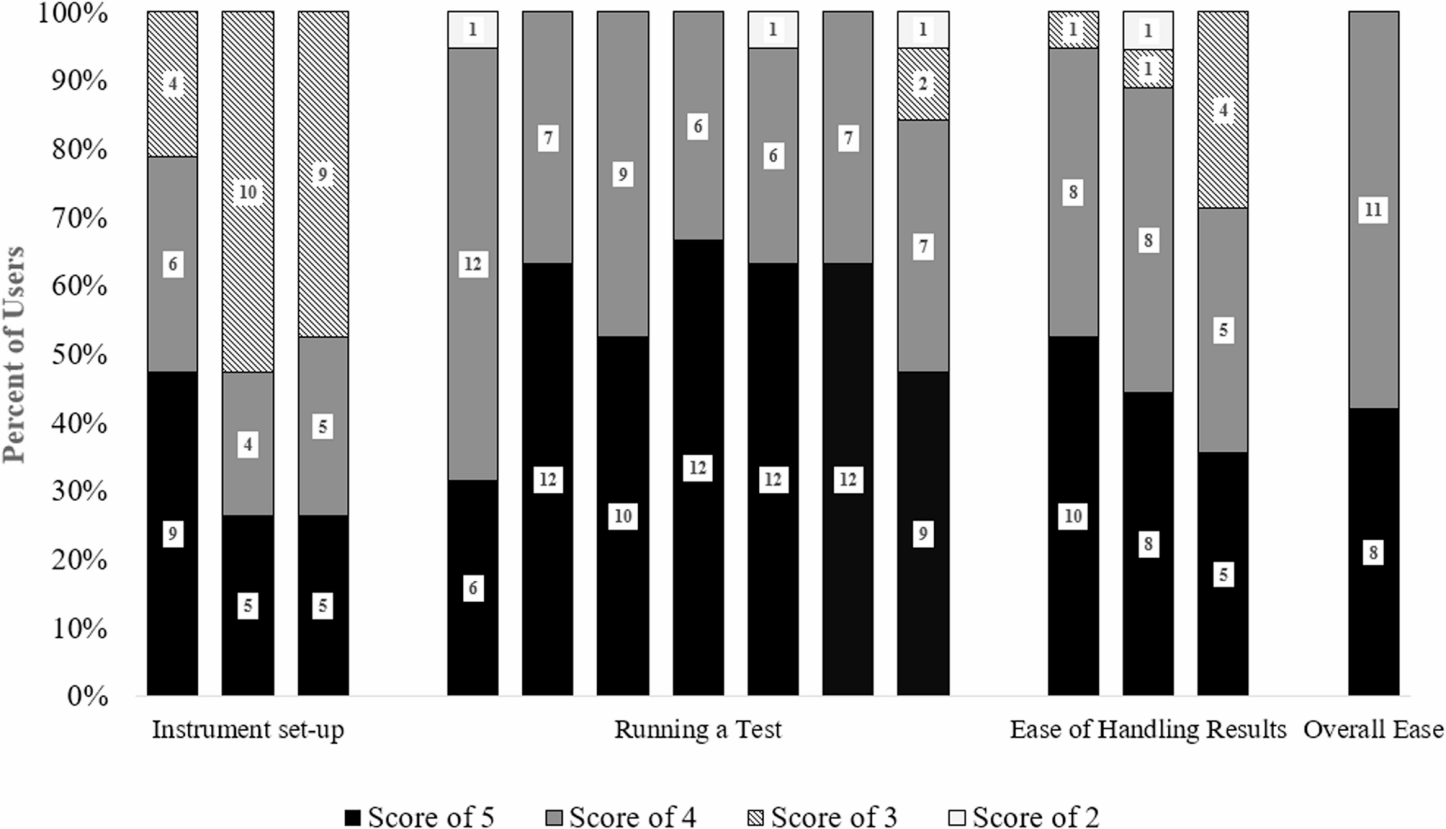
User questionnaire responses to assess instrument set-up, ease of running a test, handling results and overall ‘Ease of Use’ Based on a Likert Scale Scored from 5 (“strongly agree”), 4 (“agree”), 3 (“Neutral”), 2 (“Disagree”), 1 (“Strongly Disagree”). Data labels represent the number of users who answered each question. Of note, there were 3 questions for which the total number of users was less than 19

Users were allowed to provide a free-text commentary regarding the ease of instrument setup and ease of performing the MVP test (Table 3). Based on the user feedback on instrument setup, majority of users found the system easy to set up and use, with straightforward instructions. Based on the user feedback on ease of performing the MVP test, majority of the users found the test user-friendly and straightforward with clear instructions and helpful videos. One user suggested that error messages could be more informative, specifying the cause of the error. Overall, 19/19 (100%) of users responded that they “agree/strongly agree” that it was easy to perform the MVP test (Fig. 3).

**Table 3:** Summary of Free-Text comments from user questionnaire

Operator #	Average Score*	Q23: Please write any general comments you have regarding the ease or difficulty of setting up the Xpress system.	Q24: Please write any general comments you have regarding the ease or difficulty performing the Xpert Xpress MVP test.
1	5.0	Setting up the system was easy, instructions easy to follow.	Performing the MVP test was not complicated, very user friendly.
2	5.0	Very easy to use	No difficulty
3	3.8	Straight forward usage instructions and results. Error messages: more information would be helpful on why there was an error i.e. operator error, not enough sample provided (too little liquid solution pipetted)	
5	4.7	I had some difficulty setting up. I was unsure which parts were which and the instructions didn’t help.	Performing the Xpert Xpress MVP test was simple and the directions were easy to read. The video was helpful while performing the task.
6	3.9	Set up was a little tricky but manageable.	Performing the test was not difficult at all
7	3.9		No difficulty so much as the machine would keep saying error no reading.
11	4.4	My only comment is that I feel as though the system should be set up by both participants working on the study	Overall, the study went smoothly…it is just a little time consuming with the samples taking 1 h each.
12	5.0	The Xpress system is easy to use	
13	3.9	It would be easier if the hub came attached to the instrument. Would be better if there were more module doors to run more samples at a time.	User friendly but if the wires attached in the back were less, would be easier to set up the instrument.
14	4.1	The pipette size can make it difficult to collect specimen in the first few tries.	The test was self explanatory there is really no room for error.
15	4.0	The only difficulty I came across were the clips on the side of the monitor/screen that sits on top of the machine I knew they clipped but had to really stretch it for you to figure out it open bigger.	Testing was easy pretty straight forward.
16	4.4	Following instructions and user guide helped make it pretty seemless.	Performing the test was easy and clear when there was an error that needed to be fixed.
17	4.2	Easy when following the form. Too many buttons to power on the machine.	Running the test was easy and simple. The challenging part was getting error codes and not knowing how to navigate that or fix the machine to continue running tests.

*Average score taken from questions utilizing the Likert scale

## Discussion

In this first-ever multi-site user-experience study for POC diagnosis of vaginitis, the MVP test demonstrated consistent performance across user types (*P* > 0.082) and users’ educational status (*P* > 0.050) based on PPA and NPA estimates for all 4 targets in the MVP test. User feedback showed that 15/19 **(**79%**)** of users found the Xpress System easy to set up, 18/19 (96%**)** found the MVP test instructions easy to follow, and all 19/19 (100%) of users responded that they “agree/strongly agree” that it was easy to perform the MVP test.

Highly sensitive molecular diagnostics historically have had a long turnaround time (hours which results in a practical time for the patient of days), required expensive equipment, and required skilled operators. POC NAAT-based diagnostic tests, particularly those with CLIA-waived designation, have the potential to lessen the time from collection to patient management, to improve accuracy of same-day treatment, and to minimize long term follow up healthcare costs. The Xpert^®^ Xpress MVP test is the first CLIA-waived, FDA-cleared POC NAAT test designed to diagnose vaginal infections in symptomatic women within an hour with minimal hands-on time. Understanding the ease-of-use of these tests by operators with varying skill sets is important for effective implementation of testing strategies.

Our study was limited by the artificial nature of the testing since the performance of the assay was under evaluation and multiple comparators tests were being performed. In most cases, the tests were not performed in a manner that reflects the routine clinical workflow, which might involve a sample-first collection and testing process [5]. Fuller and colleagues found that by having patients collect samples for chlamydia/gonorrhea testing prior to engagement with a healthcare provider and beginning the test based on intake triage questions, the time to results was more offset in part by routine clinical wait times. In the clinic, they described the additional time for patients that resulted from a 90-minute test to be, on average, 46 min. Similarly, Gettinger, et al. found that a 30-minute chlamydia/gonorrhea test would add, on average, only 11 min to a visit in a very efficient clinic setting [6]. However, to utilize sample-first testing, means that the staff performing the testing may be different from the type of staff in this analysis and they may not be as comfortable with the process as the staff engaged in this research project. Additional research is needed into the implementation strategies that will best support utilization of POC tests such as the one used in this study.

Evaluation of a CLIA-waived test also impacted how new users implemented the MVP test. The absence of written competency checklist or verification of adherence to the IFU occurred prior to testing in the study reflects the practice for generalizable use and as such, our study findings may apply more broadly to the use of other POC tests as well. In contrast, users were only allowed to use the IFU and QRI for operation instructions whereas, in a real-world setting, they would have been able to contact the manufacturer for assistance.

Additionally, due to secondary nature of the analyses, the number of specimens in some of the user type categories and the educational level categories were small (large 95% confidence intervals) and hence the analyses were exploratory in nature. Thus, real-world implementation studies will be needed to assess ease of use for a variety of potential testers. This type of evaluation has been performed for chlamydia/gonorrhea testing, demonstrating the variability of performance based on the testing sites which highlights the importance of oversight and quality management to indicate when retraining or training updates may be necessary [3, 7]. Finally, it is important to note the we did not assess the cost implications of adoption because of the nature of this secondary analysis. This important factor in adoption and sustainability must be evaluated in future research efforts.

## Conclusion

The Xpert^®^ Xpress MVP could potentially fill a critical unmet need for an easy-to-use test that accurately diagnoses the most common causes of vaginitis at the site of service during an initial visit. Accurate treatment for single (which reduces over-use of antibiotics) or multiple pathogens identified by this type of testing may reduce the need for follow up visits and improve the patient experience. Xpress MVP test being the first CLIA-waived, FDA-cleared POC NAAT for diagnosing vaginal infections, has demonstrated the ease-of-use for operators with varying skill levels for effective implementation in point of care settings.

## Supplementary Information

Supplementary Material 1: Supplemental Table 1. Clinical Performance at CLIA-waived Sites in Self-Collected Vaginal Swabs.

Supplementary Material 2.

## Data Availability

All data are presented in the tables and figures in the article.
