# Supplementary material for: Evaluation of the user experience for a point of care molecular test for causes of vaginitis
Source: BMC Infect Dis. 2025 Aug 2;25:975. doi: 10.1186/s12879-025-11304-8 (PMC12318428; doi:10.1186/s12879-025-11304-8)
Supplement: Supplementary file 1 — Supplementary Material 1: Supplemental Table 1. Clinical Performance at CLIA-waived Sites in Self-Collected Vaginal Swabs. [file 12879_2025_11304_MOESM1_ESM.docx]

Supplemental Table 1. Clinical Performance at CW Sites in Self-Collected Vaginal Swabs

| **Target** | | **Positive** | **Positive Agreement (N)** | **Positive Agreement (%)** | **95% CI** | **Negative** | **Negative Agreement (N)** | **Negative Agreement (%)** | **95% CI** |
| --- | --- | --- | --- | --- | --- | --- | --- | --- | --- |
| **BV** | **Total** | 464 | 434/464 | 93.5 | 90.9 – 95.4 | 760 | 711/7610 | 93.6 | 91.6 – 95.1 |
| **TV** | **Total** | 137 | 131/137 | 95.6 | 90.8 – 98.0 | 1191 | 1188/1191 | 99.7 | 99.3 – 99.9 |
|  | **Fresh** | 48 | 47/48 | 97.9 | 89.1 – 99.6 | 1162 | 1159/1165 | 99.7 | 99.2 – 99.9 |
|  | **Contrived*** | 89 | 84/89 | 94.4 | 87.5 – 97.6 | 29 | 29/29 | 100 | 88.3 – 100 |
| **CS** | **Total** | 367 | 359/367 | 97.8 | 95.8 – 98.9 | 865 | 804/865 | 92.9 | 91.0 – 94.5 |
| **CgCk** | **Total** | 132 | 131/132 | 99.2 | 95.8 – 99.9 | 1227 | 1222/1227 | 99.6 | 99.1 – 99.9 |
|  | **Fresh** | 33 | 33/33 | 100 | 89.6 – 100 | 1199 | 1195/1199 | 99.7 | 99.1 – 99.9 |
|  | **Contrived*** | 99 | 98/99 | 99.0 | 94.5 – 99.8 | 28 | 27/28 | 96.4 | 82.3 – 99.4 |
