# Supplementary material for: Evaluation of the user experience for a point of care molecular test for causes of vaginitis
Source: BMC Infect Dis. 2025 Aug 2;25:975. doi: 10.1186/s12879-025-11304-8 (PMC12318428; doi:10.1186/s12879-025-11304-8)
Supplement: Supplementary file 2 — Supplementary Material 2. [file 12879_2025_11304_MOESM2_ESM.pdf]

Rx only IVD

Cepheid. A better way.

Quick Reference Instructions

Xpert® **Xpress** MVP and the GeneXpert® **Xpress** System (Hub configuration)

For *In Vitro* Diagnostic Use. CLIA Complexity: Waived. A Certificate of Waiver is required to perform this test in a CLIA Waived setting*.
For use with vaginal swab specimens.

Before you begin

- Read through the entire Quick Reference Instructions before beginning a test.
- Start the test within 30 minutes of adding the specimen to the cartridge.
- Instructions on how to prepare the specimen and the cartridge are shown in a video within the software and in these Quick Reference Instructions.
- The recommended environmental operating conditions for Xpert **Xpress** MVP are 15–30 °C, 20–80% relative humidity.
- Only use transfer pipettes provided in the Xpert Xpress MVP test kit.

Storage and Handling

- Wear gloves. Change gloves between processing each sample.
- Store the Xpert **Xpress** MVP cartridges at 2–28 °C until the expiration date provided on the label.
- Specimens should be transported at 2–28 °C.
- Specimens placed in transport medium following collection can be stored for up to 42 days at 2–28 °C prior to testing with the Xpert **Xpress** MVP test.
- **DO NOT** open or alter any part of the **used** cartridge for disposal.

1 Patient Specimen Collection

Swab specimens must be collected by trained personnel or self-collected in a clinical setting and transported with the Xpert Swab Specimen Collection kit (catalog number SWAB/G-50-US). Refer to specimen collection instructions provided with the collection kit.

2 Starting the System

A Starting the Software

- Put on a clean pair of gloves
- Turn on the GeneXpert **Xpress** instrument, and the Hub computer
- The Windows lock screen appears. Swipe up to continue
- Enter the Windows password
- Allow 30 seconds for the **Xpress** software to load

B Enter UserID and Password

- Touch the **User Name** field to display the virtual keyboard
  - Enter your user name and password
  - Touch the **X** in the upper right of the virtual keyboard

C Or, Use Barcode Scanner Shown Below to Scan Employee Badge or User Login Barcode

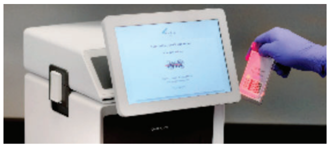

D Home Screen Displays

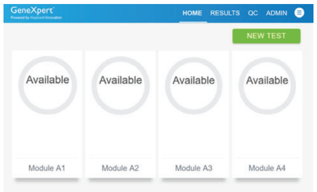

3 Testing a Patient Specimen

WARNINGS

- **DO NOT** use a cartridge that is wet or has leaked.
- **DO NOT** use a cartridge that has been dropped.
- **DO NOT** open a cartridge lid until you are ready to perform testing.
- **DO NOT** shake or tilt the cartridge after adding the sample.
- **DO NOT** reuse disposable pipettes or cartridges.
- **DO NOT** turn off or unplug the instrument while a test is in progress as this will stop the test.
- **DO NOT** use a cartridge that has a missing or damaged reaction tube.

Refer to the Instructions for Use for Warnings and Precautions, Specimen Collection and Handling, and Quality Control Testing.

3 Testing a Patient Specimen (continued)

A Start a Test

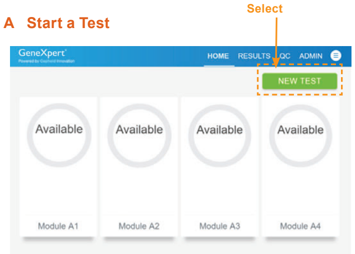

B

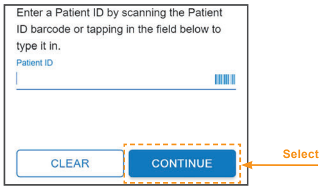

C Confirm patient information as it can not be altered after submission

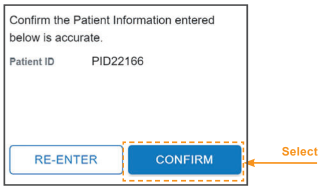

D

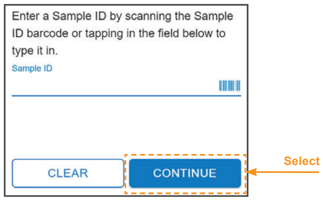

E

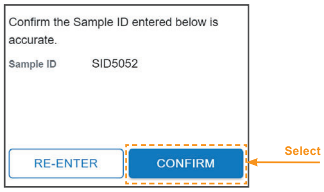

F Scan Cartridge Barcode

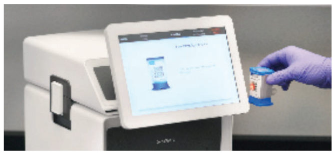

3 Testing a Patient Specimen (continued)

G Enter your user name and password if prompted, then:

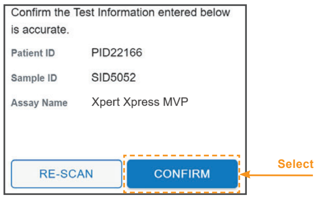

H Watch video & touch **CONTINUE** when complete

I Open cartridge lid

J Check that the specimen tube is fully capped and **vigorously shake the transport tube 3–4 times**

K Open tube lid

L Fill Pipette with Sample

- Obtain a new transfer pipette provided in the Xpert Xpress MVP test kit
- Squeeze the top bulb of the pipette **completely until it is fully flat**. While continuing to hold the bulb fully flat, place the pipette tip in the specimen transport tube.
- Keeping the pipette below the surface of the liquid, release the top bulb of the pipette **slowly until the pipette is completely filled with sample** before removing it from the tube. It is okay if liquid goes into the overflow reservoir.

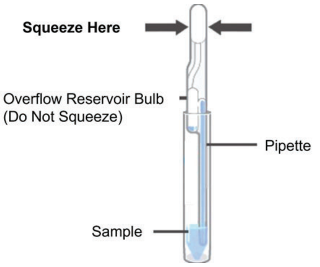

M Transfer Sample to Cartridge

- Put the pipette into the large opening on the lower right corner of the cartridge (Sample Chamber). Squeeze the top bulb of the pipette **completely until it is fully flat** to empty the contents. Some liquid may remain in the overflow reservoir.

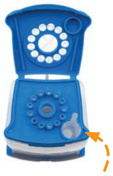

Sample Chamber (Large Opening)

- **Continue to hold the top bulb fully flat and do not release** until the pipette is removed from the cartridge. **Do not reuse a pipette.** Dispose of the used pipette.
- Close the cartridge lid. After cartridge is prepared, touch **CONTINUE** on Cartridge Preparation screen.

3 Testing a Patient Specimen (continued)

N Load Cartridge Into Module

- Pull open the instrument door with the flashing green light
- Load the cartridge
- Close the door until it clicks
- The **Test Loading** screen will appear followed by the **Test Running** screen in less than a minute
- If performing a new test, go to Section 4 below.

O Results

- When the test is completed, the **Test Completed** screen appears with the test results and the instrument door will unlock.
- Or, if on the **HOME** screen, touch the **RESULTS** button to view test results.
- Confirm the test provided a positive or negative result. If **NO RESULT**, repeat test with a new cartridge.
- To print report, touch the print icon.
- Remove cartridge. Dispose of the cartridge and gloves according to your institution's policy. Go to Section **4 'How to Start a New Test While a Test is Running'** if performing a new test.
- To log out, touch the User icon then select **LOGOUT**.

4 How To Start a New Test While a Test is Running

- Touch the **HOME** button on the Test Running screen
- The **HOME** screen will display the module status and the **NEW TEST** button
- You can now perform the same steps in Section **3 'Testing a Patient Specimen'** to perform a new test. Change gloves between processing each sample.

5 Possible Results

For Patient Specimen or External Control

Results Interpretation

BV NEGATIVE
Negative test for Bacterial Vaginosis (BV).

Candida group NOT DETECTED
Candida group target DNA is not detected.

Candida glab-krus NOT DETECTED
Candida glabrata/Candida krusei target DNA is not detected.

TV NOT DETECTED
Trichomonas vaginalis (TV) target DNA is not detected.

BV POSITIVE
Positive test for Bacterial Vaginosis (BV).

Candida group DETECTED
Candida group target DNA is detected.

Candida glab-krus DETECTED
Candida glabrata/Candida krusei target DNA is detected.

TV DETECTED
Trichomonas vaginalis (TV) target DNA is detected.

NO RESULT - REPEAT TEST
If the result is **NO RESULT - REPEAT TEST**, then retest with a new cartridge using a new transfer pipette.

5 Possible Results (continued)

Results Interpretation

INSTRUMENT ERROR
Result is an instrument error. Touch **CLEAR ERROR** and follow the on-screen instructions. When the Home screen appears, repeat the test using a new cartridge and a new transfer pipette.

NOTE: If an incorrect result is provided for the external control, repeat the external control run.

6 How to View Status of Tests In Progress and Completed Tests

A Touch HOME button

- To view the status of tests in progress or completed tests.

B View Test In Progress Above the Module Number

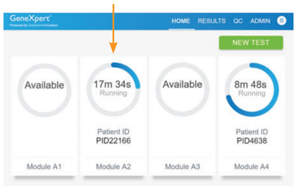

C View Completed Tests

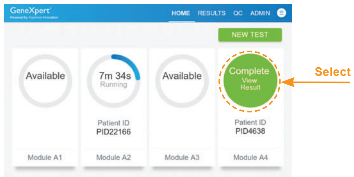

NOTE: Refer to the Instructions for Use for information on reviewing or printing past results.

7 How to Run External Controls – Positive and Negative Controls

Note: The GeneXpert **Xpress** system must have software version 6.2 or higher to use the Xpert **Xpress** MVP test.

The following external controls are recommended for use with Xpert **Xpress** MVP:

- NATtrol™ Vaginal Negative Control, ZeptoMetrix Corporation catalog number NATVNEG-6C
- NATtrol™ Vaginal Positive Control, ZeptoMetrix Corporation catalog number NATVPOS-6C

It is recommended that external controls be tested at the frequency noted below.

- Each time a new lot of Xpert **Xpress** MVP kits is received.
- Each time a new shipment of Xpert **Xpress** MVP kits is received even if it is the same lot previously received.
- Each time a new operator is performing the test (i.e., operator who has not performed the test recently).
- When problems (storage, operator, instrument, or other) are suspected or identified.
- If otherwise required by your institution's standard Quality Control (QC) procedures.

7 How to Run External Controls – Positive and Negative Controls (continued)

A On the Home screen or the Test Running screen, touch QC

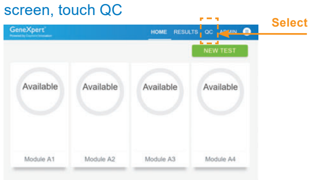

B Select the QC list option based on the test you are performing

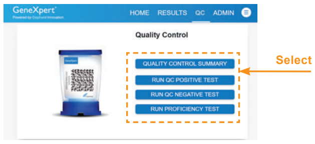

C Type **Positive Control** or **Negative Control** or scan Sample ID barcode.

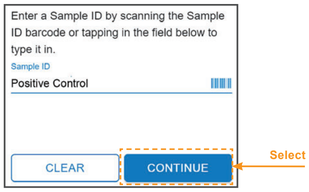

D

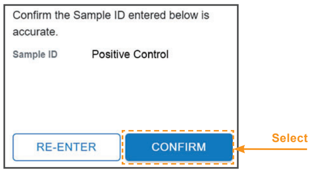

E Scan Cartridge Barcode

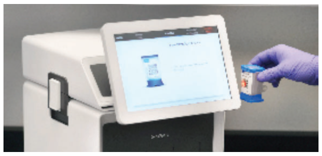

F

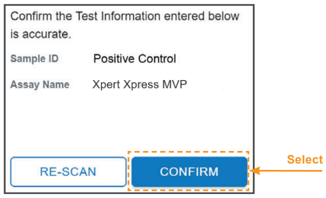

G

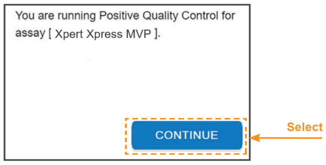

H Proceed with steps **H-O** from the above Section **3 'Testing a Patient Specimen'**, this time for "quality control" instead of "specimen".

* CLIA Waiver: To obtain CLIA waiver information and a Certificate of Waiver, please contact your state health department. Additional CLIA waiver information is available at the Centers for Medicare and Medicaid website at www.cms.hhs.gov/CLIA. Failure to follow the instructions or modification to the test system instructions will result in the test no longer meeting the requirements for waived classification.

Rx only | IVD | *In Vitro* Diagnostic Medical Device

CORPORATE HEADQUARTERS

904 Caribbean Drive
Sunnyvale, CA 94089 USA
TOLL FREE +1.888.336.2743
PHONE +1.408.541.4191
FAX +1.408.541.4192

www.**Cepheid**.com

Cepheid
904 Caribbean Drive
Sunnyvale, CA 94089
USA

TECHNICAL SUPPORT

TOLL FREE +1.888.838.3222
EMAIL techsupport@cepheid.com
www.cepheid.com/en_US/support/contact-us

© 2023 Cepheid
